# A Node Density Control Learning Method for the Internet of Things

**DOI:** 10.3390/s19153428

**Published:** 2019-08-05

**Authors:** Shumei Lou, Gautam Srivastava, Shuai Liu

**Affiliations:** 1 College of Mathematics and Computer Science, Xinyang Vocational and Technical College, Xinyang 464000, China; 2 Department of Mathematics & Computer Science, Brandon University, Brandon, MB R7A6A9, Canada; 3 Research Center for Interneural Computing, China Medical University, Taichung 40402, Taiwan; 4 College of Computer Science, Inner Mongolia University, Hohhot 010012, China; 5 Hunan Provincial Key Laboratory of Intelligent Computer and Laungrage Information Processing, Hunan Normal University, Changsha 410081, China

**Keywords:** Internet of Things, wireless sensors, mobile nodes, density control, probability

## Abstract

When examining density control learning methods for wireless sensor nodes, control time is often long and power consumption is usually very high. This paper proposes a node density control learning method for wireless sensor nodes and applies it to an environment based on Internet of Things architectures. Firstly, the characteristics of wireless sensors networks and the structure of mobile nodes are analyzed. Combined with the flexibility of wireless sensor networks and the degree of freedom of real-time processing and configuration of field programmable gate array (FPGA) data, a one-step transition probability matrix is introduced. In addition, the probability of arrival of signals between any pair of mobile nodes is also studied and calculated. Finally, the probability of signal connection between mobile nodes is close to 1, approximating the minimum node density at T. We simulate using a fully connected network identifying a worst-case test environment. Detailed experimental results show that our novel proposed method has shorter completion time and lower power consumption than previous attempts. We achieve high node density control as well at close to 90%.

## 1. Introduction

With the rapid development of modern technology, Internet of Things (IoT) computing integrates multiple discipline technologies such as modern sensors, microelectronics, communication, embedded computing, and distributed information processing. It has also attracted worldwide attention and gradually has been integrated into people’s daily lives [1]. In IoT, wireless sensor networks (WSNs) are inexpensive to install and easy to use, thus leading to their popularity and use. Therefore, it is applied in practical applications of IoT where it is inconvenient or necessary to eliminate wired connections and is widely used in many fields. These include military, medical, healthcare, chemical processing, and disaster relief [2]. Faced with many practical applications, how can implementers ensure the connectivity of an IoT network and maximize the lifetime of the long network? Sensor nodes were generally randomly distributed in places with harsh natural environments. We can observe two extremes under these environmental conditions. First, when the density of the nodes is too large, the communication conflicts between the nodes increased. Moreover, when the density of the nodes is too small, the connectivity of the system and the coverage of the monitored area cannot be guaranteed. So we live in a paradoxical situation trying to find the ideal number of nodes. The quality of service and network power consumption of WSNs are directly affected by the density of nodes [3,4]. Relevant scholars have proposed some better methods.

### 1.1. Related Work

In some early work, an optimized coverage control method for joint-aware wireless sensors was proposed in [5]. The solution process of maximum seamless coverage when three-node joint coverage was given in this method. On the one hand, the method for solving the node coverage quality expectation and the coverage determination method when compared to neighbour nodes was verified by the probability correlation knowledge when the sensor nodes were covered in the monitoring area. On the other hand, when there was redundant coverage, a scaling factor was introduced to complete the calculation process of redundant coverage when any sensor node was in redundant node coverage, thereby completing the control. This method can effectively suppress the rapid consumption of node energy. However, the control completion time was too long.

We have also seen a WSN monitoring node grouping control method based on evolutionary game theory proposed in [6]. Firstly, the non-cooperative game model was constructed with the utility function of the game as the objective function by mapping the search space of the optimal node set to the policy combination space of the game. Secondly, the target optimization was realized by the Nash equilibrium analysis and the balanced disturbance recovery process; Then, the clustering algorithm was designed to optimize the node set to form the corresponding group to participate in the final positioning; Finally, the control was done based on the final positioning result. The method avoided the problem of high energy consumption of nodes in the common coverage area of multiple radiation sources, however, the control power consumption was large.

In [7], a multi-source fault-tolerant topology node control method in a WSN was proposed. In a heterogeneous wireless sensor actuator network model consisting of a large number of computational, energy-constrained sensor nodes and a small number of better-performing actuator nodes, any sensor nodes must be guaranteed and executed to minimize the total path power consumption. There were at least k disjoint paths between nodes, at the same time; the nodes with better weights were selected. In this way, the connectivity of the network was not affected when any k−1 nodes fail, thereby effectively completing the node control. However, the time taken for the method to control the completion was too long.

Recently, a three-dimensional WSN topology control method based on node degree estimation was proposed in [8]. According to the node degree factor of the degree of topology change, the node degree estimation model for evaluating the comprehensive performance of the network was constructed in this method. At the same time, the topology control algorithm based on the model was proposed. Topology creation and optimization were achieved by arranging sensor nodes, creating network topologies, generating data transmission links, and correcting node transmit power. The method had a better overall performance of topology control; however, the control power consumption was large. Therefore, a density control method of self-learning based wireless sensor mobile node is proposed here and applied to the Internet of Things.

### 1.2. Paper Organization

The rest of the paper is organized as follows. In Section 3, characteristics of WSNs under IoT environments are provided. Next, Section 4 provides the composition of mobile nodes. Section 5, we design the learning algorithm of node density control. Experimental results show that our proposed method has a shorter completion time and and lower power consumption high node density control accuracy in Section 6. Finally, Section 7 concludes our work.

## 2. Overview of Node Density Control

In this section we give an overview of node density control. In general, the number of nodes in WSNs is large and the distribution of those nodes is very dense. The nodes are prone to failure when they are affected by environmental control or run out of available battery life. Environmental interference and node failure can easily cause changes in the network topology from its initial deployment. In this case, multiple nodes work at the same time to reduce the working load on any single node. In order to extend the life of a single node, a rotation cycle is usually implemented to reduce energy consumption and balance the energy of nodes. Therefore node density within the network itself needs to be controlled. Therefore, density control is allowing some nodes into a low-power sleep state without sacrificing system performance, while keeping only some nodes as active working nodes. In this way, the density of active nodes in the network can be reduced. A by product of effective node density control is that the redundancy of perceived data can be reduced and wireless communication conflicts and interference can be reduced as well. We also observe a reduction of energy consumption of the whole system. When a work node fails due to a dead battery or other reasons, neighbouring sleep nodes can resume work to replace the failed node and continue to maintain the normal workload of the network. Therefore, a well schemed density control system can effectively prolong the lifetime of a WSN. Its study and effective strategies are crucial to effectice WSN and IoT as a whole.

## 3. Characteristics of Wireless Sensor Networks

The sensory factors used by WSNs to sense the real world under IoT are composed of a large number of low-cost nodes with a certain organizational structure [9]. It collects, processes, communicates and identifies environmental sensing variables through inter-working. To improve the effect of density control of mobile wireless sensor node, one needs to first analyze the characteristics of the WSN, with its unique characteristics of WSN node structure is analyzed, which uses the geometric model for road use attenuation node density and node connection probability and so on, in order to realize the control of node density. Specific characteristics of WSNs include:

- autonomous networking
- large-scale applications
- short-range communication
- multi-hop routing
- node free distribution
- multi-point sensing of data
- network adaptive topology
- low power consumption
- low cost
- limited communication and processing capabilities
- diversified application scenarios
- self-healing compensation
- remote monitoring and control

The line sensor network is a peer-to-peer autonomous network with strong applicable background. The network structure and communication protocol have great differences according to different application scenarios. The node topology control process changes according to specific needs.

WSNs used in IoT environments have some common features due to their many limitations.

1. Limited hardware resources
  WSNs generally require low cost and low power consumption of nodes. Therefore, no matter the distance and bandwidth of communication, the collection, and processing of information, or the security and reliability of data, the hardware resources of nodes are quite limited. Making the most of limited resources based on specific applications is a priority for every wireless sensor design. In particular, for a large number of multi-node applications, the resource allocation of nodes not only causes a huge difference in cost but also has a significant impact on the communication quality of the entire network. Limited resource allocation needs to integrate the influence of various aspects of the whole network, including network topology, data collection rate, routing communication protocol, data security assurance mechanism to fully analyze the optimal allocation scheme of nodes.
2. Adaptive network topology
  In the application of WSNs, each node in a large number of nodes may have position changes, node damage, online networking failure, communication link quality deterioration, and error data packet delivery. When the topology of a wireless communication network has a small range or a very low probability of a large-scale change, the reliability of communication can be greatly affected. The WSN itself should have the ability to adapt or reorganize the network, restore the network topology and multi-hop mechanism within a certain period of time, and ensure that the network jumps back to a normal working state. The adaptive capability of the WSNs has high requirements on the data link layer protocol, the medium access layer protocol and the routing protocol used by the network, and the networking function of the network is completed with a low network overhead.
3. Multi-hop routing protocol
  The information collected by the network node needs to be transmitted through the relay of the surrounding nodes and selectively transmitted to the aggregation node in multiple ways so that the data can complete the final summary, display, and networking. The routing protocol between nodes determines the communication quality and service life of the network. Different network applications use different routing protocols and the multi-hop routing mechanism is also very different. However, finally, the data of the collection node needs to be transmitted to the aggregation node through the multi-hop route.
4. Self-awareness and ability to repair
  The nodes of WSNs are composed in various forms, the distribution is freely dispersed, and the life and use of nodes can be different. This causes the fading coefficients of different regions of the network to be different, and some areas of the network will have empty holes earlier, which may make the network function incomplete or even fragmented. The WSN node should have the capability of self-aware repair, timely report and report its own state, and be used to adjust the routing mechanism and supplement the network topology relationship in time. WSNs require long-term battery life. In addition to their low power consumption and high use rate, the network itself should have an overall adjustment function to balance the aging rate of the network, which is a must for WSNs.
5. Remote monitoring control
  Remote monitoring control means that the aggregation node in the network can be located anywhere in the network, collect and upload data to the network, control various working modes of the network, and adjust network operating parameters in real time. The aggregation node in the WSN has high flexibility and is the core control node of the entire network. There is a high data communication rate around the aggregation node. Therefore, there is a high requirement for the use of the channel.

## 4. Analysis of Mobile Node Structure in Wireless Sensor Networks

Wireless sensor nodes use a mobile node to traverse the whole network and periodically broadcast information containing their current location. Therefore, in order to achieve node density control, it is necessary to analyze the specific structure of mobile nodes. Based on the above analysis of the characteristics of WSNs give in Section 3, the structure of mobile nodes in WSNs is analyzed. The node structure can be used to inspect the distribution of nodes and obtain the node density, so as to control the node density.

The wireless network sensor node structure consists of two parts, including a wireless RF transceiver module designed with the CC2430 chip as the core and an FPGA development board used as a real-time data processing module (Model Spartan-3E-based by Xilinx) 500E, with 500,000 system doors) [10]. The wireless radio frequency module is mainly composed of CC2430 chip and serial port driver chip SP3223E, which is used to complete network wireless interconnection and data transceiving work.The FPGA development board uses the embedded CLinux system to implement data exchange with the wireless transceiver module through the RS232 serial port after completing the hardware configuration and software development of the system [11].

The circuit design of the wireless RF transceiver module uses the CC2430 chip of the Zig-Be protocol. The CC2430 uses its own MCU (the 8051 uses the Carrier Sense Multiple Access/Collision Prevention (CSMA/CA) method to communicate with other network nodes and exchange the required information and data according to the Zigbee protocol.

The CC2430 communicates with the serial port of the FPGA development board through the serial port driver chip SPE3223E. The serial port initialization is first performed before the start of the work, including setting the clock control register CLKCON, and selecting the clock operating frequency to be 32 MHz Besides, clear the UOCFG bit of the peripheral device register PER-CFG, select USARTO to position 1, select the function of the PO port by the bit operation instruction POSEL=OxOC, and select the PO port function selection register POSEL 2, 3 Position 1. which is set to the external device function and is consistent with the pins used in the hardware circuit design.

Equation (1) is given by the baud rate generator integrated into the CC2430 chip.

(1) $$Baudrate=\frac{\left(256+BAUD\_M\right)\times 2^{BAUD}}{2^{28}}\times F$$

In Equation (1), the baud rate is obtained by writing the desired value to the BAUD_M[7:0] bits of the Baud Rate Control Register UOBAUD and the BAUD_E[4:0] bits in the General Control Register obtaining the desired transmit and Receive rate. In Equation (1), *F* represents the serial communication rate.

The serial port receiving and transmitting work uses the interrupt completion. The setting of the serial port working mode is completed by setting the USARTO control and status register UOCSR and the UART control register UOUCR. It sets USARTO to UART mode, URXOIE is set to 1, the USART transceiver status is activated and the USARTO stop bit level is set high. The data transceiver work is completed by the interrupt function, which is called when the serial port has data to send and receive. Instruction of interrupt function in registration is defined as Equation (2).

(2) $${}^{\#}pragmavector=\frac{1}{Baudrate}\left(USARTO★UART\right)$$

The program is written in the function body to complete the transmission and reception of serial data through the USARTO’s receive/transmit buffer register UOBUF. Finally, the EAL bit of the interrupt control register IENO is set to 1 to allow the corresponding interrupt to occur in the system [12].

The setting of the radio frequency module part is no different from the normal case. After the corresponding programming, the RF data antenna transmits its own information data and acquires the data information of other network nodes, and the obtained data is transmitted to the FPGA through the serial port or the processed data is obtained from the FPGA. The entire system program is executed after the CC2430 is set up [13].

The module can also be used alone as a network coordinator, router in a wireless network or as a simple application device.

## 5. Design of Node Density Control Learning Algorithm

Based on the structure characteristics of mobile nodes in WSNs, we design a novel node density control learning algorithm. The road consumption decay geometry model [14] is used in the algorithm design. Assuming that each node has the same transmit power *t* and receive threshold power *m*, the receive decibel threshold of the sensor node obtained by the transmit power and the receive threshold power is *h*, then the signal-to-noise decay is defined as Equation (3).

(3) $$\beta_{1}\left(u,v\right)=10\times h\mathrm{lg}s\left(u,v\right)$$

Set the frequency to $\beta_{th}$. If $\beta_{1}\left(u,v\right)\leq \beta_{th}$, there is an edge between the two nodes, otherwise there is no edge between the two nodes. When $\beta_{1}\left(u,v\right)=\beta_{th}$, the obtained s(u,v) is the maximum one-hop communication radius of the node *u*, which is denoted as $r_{0}$. Because the model does not consider the interference of the obstacle and the surrounding environment of the site to the node channel, the model has a large error. Yang et al. designs a more practical and accurate time attenuation model based on the geometric model of road loss [15]. The interference of various bad factors in the network to the communication ability of the node is characterized by a random variable obeying the normal distribution, and the probability density function is given in Equation (4).

(4) $$f\left(\beta_{2}\right)=\frac{1}{2\pi \sigma }\exp\left(-\beta^{2}/\left(2\sigma^{2}\right)\right)$$

In Equation (4), β represents the characteristics of density distribution, and σ represents probability coefficient.

Then, the signal-to-noise decay formula can be defined as Equation (5).

(5) $$\beta \left(u,v\right)=\beta_{1}\left(u,v\right)+f\left(\beta_{2}\right)$$

If $\beta_{1}\left(u,v\right)+\beta_{2}\leq \beta_{th}$, the two-node *u* and *v* can establish the side of direct communication. The probability of having an edge between two nodes is defined as Equation (6).

(6) $$P\left(\Lambda \left(u,v\right)\left|s\right.\left(u,v\right)\right)=P\left(\beta \left(u,v\right)\leq \beta_{th}\left|s(u,v)\right.\right)$$

Substituting the probability density function of the variable $\beta_{2}$ into Equation (6), Equation (6) can be simplified to obtain Equation (7).

(7) $$P\left(\Lambda \left(u,v\right)\left|s\right.\left(u,v\right)\right)=\frac{1}{2}-\frac{1}{2}erfP\left(\frac{10a}{\sqrt{2\sigma }}1g\frac{s(u,v)}{r_{0}}\right)$$

The number of neighbors of a node is called the degree of the node, and its mean is defined as in Equation (8).

(8) $$E\left(D\right)=\sum_{i=1}^{\infty }P_{i}\left(\Lambda \left|s_{i}\right.\right)\pi {\left(s_{i}^{2}-s_{i-1}^{2}\right)}_{\rho }$$

In Equation (8), ρ represents node density.

Simplify Equation (8) to obtain Equation (9).

(9) $$E\left(D\right)=2\pi_{\rho }\int_{0}^{\infty }P\left(\Lambda \left|s\right.\right)sds+0$$

The mobile node degree is a random variable obeying the Poisson distribution with an intensity of E(D). Using the properties of the Poisson distribution, the probability that a node is an isolated node can be defined as Equation (10).

(10) $$P\left(D=0\right)=e^{-E(D)}$$

In an infinitely large area or a closed spherical area, the WSN is connected equivalent to no isolated nodes in the network. However, the two are not equivalent to a limited area or a non-closed spherical area. The conditions of no isolated nodes in the network are weak, and the conditions under which the network is connected are strong [16]. As the density of nodes increases, the network first reaches the state of no isolated nodes, and further increases the node density to reach the fully connected state [14]. In a limited or un-closed area, you can first find the node density ρ when the network reaches the state of no isolated nodes. Based on this, the one-step transition probability matrix of the signal is used to find the sooner or later probability f(u,v) of the signal mutual access between any pair of nodes, and it is close to 1 to approximate the node density $\rho^{*}$ when the network is fully connected. $P\left(noisolation\left|number\;of\;nodes\;is\;n\right.\right)={\left(1-e^{-E(D)}\right)}^{n}$ can be found as the mean of the average number of neighbors of a given node. Therefore, the probability of no isolated nodes in the network is defined as the unclosed region of Equation (11) with an area of *A* and a node density of ρ.

(11) $$\begin{array}{cc} P(noisolation) & =\sum_{n=0}^{\infty }P\left(N-n\right){\left(1-P\left(D=0\right)\right)}^{n}\\ & =\sum_{n=0}^{\infty }\frac{{\left(\rho A\right)}^{n}}{n}e^{-\rho A}\times {\left(1-P\left(D=0\right)\right)}^{n}\\ & =e^{-\rho A}\sum_{N=0}^{\infty }\frac{{\left(\rho A-\rho AP\left(D=0\right)\right)}^{n}}{n} \end{array}$$

The probability *P* is used to represent the density ρ of the mobile node, *n* is the number of nodes, and *N* is one-step transition probability matrix. We observe that when Equation (11) removes the logarithm on both sides, we obtain Equation (12).

(12) $$InP=-\rho Ae^{-E(D)}=-\rho Ae^{-2\pi \rho \xi }$$

where ξ represents regional closure index. Simplify Equation (12) to obtain Equation (13).

(13) $$\frac{2\pi \xi }{-A}InP=2\pi \rho \xi e^{-2\pi \rho \xi }$$

When x=2πρξ, set the inverse function of the function $y=xe^{-x}$ to x=g(y), substituting it into Equation (13) to obtain Equation (14).

(14) $$\rho =\frac{1}{2\pi \xi }g\left(\frac{2\pi \xi InP}{-A}\right)$$

In Equation (14), $\xi =\int_{0}^{\infty }P\left(\Lambda \left|s\right.\right)sds$. When the probability *P* of no isolated nodes in the network is close to 1, the corresponding node density ρ can be obtained by Equation (14). The number of nodes on the area *A* is *n*. Each node uses its own positioning device to locate and transmit information to the base station. For each node *u*, the base station calculates the distance S(u,v) between the node and the other n−1 nodes by using the position information of each node and substitutes Equation (7) to obtain the probability that the node *u* communicates directly with the other n−1 nodes. The probability of getting the signal jumping from node *u* to node *v* is Equation (15).

(15) $$N\left(u,v\right)=\frac{P(\Lambda \left(u,v\right)\left|S(u,v)\right.)}{\sum_{v=u}P\left(\Lambda \left(u,v\right)\left|S(u,v)\right.\right)}$$

Establish a one-step transition probability matrix *N* for n×n order. Let Equation (15) be used as the element at the intersection of the row corresponding to the node *u* and column of node *v* in the matrix *N*. Each node is considered to be a state of the signal. When a signal is transferred from one node to another, it can be seen as a signal being transferred from one state node to another. The Markov property of a signal moving from one state node to another is discussed below.

Corresponding to the node density ρ, the process of transmitting a signal in its generated WSN can be seen as a finite, homogeneous Markov chain [15].

With each node as a state of the signal, the number *n* of nodes is a finite number. The signal then has *n* states for its transfer, which means that the state set of signal transitions is finite and discrete. At the same time, the signal is transferred from one state node to another, and the number of hops passing through is an integer, that is, its parameter set is also discrete. Since the probability $P(\Lambda \left(u,v\right)\left|S(u,v)\right.))$ of the signal transitioning from the state node *u*to the state node *v* is independent of the state node experienced before the signal is transmitted to the state node *u*, and is also independent of the number of hops experienced before the signal is transmitted to the state node *u*, it is only related to the signal currently related to this condition in the state node *u*. Therefore, the process of transmitting signals in this WSN is a finite, homogeneous Markov chain.

When the WSN corresponding to the node density ρ generation is a connected network, each state node is in a constant return state. The process of transmitting signals in the network at this time can be regarded as a finite, homogeneous, irreducible Markov chain.

For any two state nodes *u* and *v* in a finite, homogeneous Markov chain, the late-early probability that the signal moves from the state node to the state node is defined as Equation (16).

(16) $$f\left(u,v\right)=\lim\frac{\sum_{e=1}^{E}N^{e}\left(u,v\right)}{1+\sum_{e=1}^{E}N^{e}\left(u,v\right)}$$

In Equation (16), $N^{e}\left(u,v\right)$ represents the probability that the signal *e* step is transferred from the state node *u* to the state node *v*.

If the WSN corresponding to the density ρ generation is disconnected, there is a state node pair (u,v), and the sooner or later probability that the signal is transferred from the state node *u* to the state node *v* satisfies f(u,v)→0. If the network is connected, then for any state node pair (u,v), there is f(u,v)→1.

If the WSN generated corresponding to the node density ρ is not connected, there are at least two connected branches in the network. The state nodes *u* and *v* are respectively taken out from different connected branches, and the two state nodes are disconnected. That is $P(\Lambda \left(u,v\right)\left|S(u,v)\right.))\to 0$, there is N(u,v)→0. Because the state nodes *u* and *v* belong to different connected branches respectively, after any *e* step, the two state nodes are not connected, then there is $N^{e}\left(u,v\right)\to 0$. Bring it into Equation (16) to get f(u,v)→0. If the WSN corresponding to the node density ρ is connected, it can be seen from the above that the transmission process of the signal in the network can be regarded as a finite, homogeneous and irreducible Markov chain [16,17]. It can be found f(u,v)→1 by the Doeblin formula.

The algorithm description is as follows.

First, we use Equation (14) to find the node density ρ when there is no independent node corresponding to the actual network. Then, the WSN corresponding to the density ρ is randomly generated in a normal distribution. Each node locates with its own positioning device and transmits the location information to the base station. The base station calculates the signal one-step transition probability matrix *N* using the position information of each node. In addition, by Equation (16), the sooner or later probability that the signal state node *u* is transferred to the state node*v* is obtained. If the early or late probability between any two state nodes is close to 1, then the network is connected at this time, otherwise, the node’s delivery density is increased. Each newly added state node performs its own positioning by using its own positioning device, and transmits data to the base station, and the base station obtains the mutual communication probability $P(\Lambda \left(u,v\right)\left|S(u,v)\right.))$ between each state node pair. At the same time, the one-step transition probability matrix N(u,v) of the signal between each state node pair is corrected. Then, Equation (16) is used again to find the late-to-earth transition probability f(u,v) of the signal between the pairs of nodes and to judge the connectivity of the network. Loop through the above steps until the probability approaches the node density when the network is connected.

If you want to ensure that the sensor network is connected, you only need to ensure that all $\left[N-(k-1)\right]$-order sub-matrices of the signal one-step transition probability matrix *N*should satisfy the following properties. The late-early probability f(u,v) of the state node pairs contained in the order-sub-matrix of the one-step transition probability matrix is close to 1. Based on this property, the minimum node density of the WSN that is *K*-connected can be found.

## 6. Experimental Results

**Figure 1:**
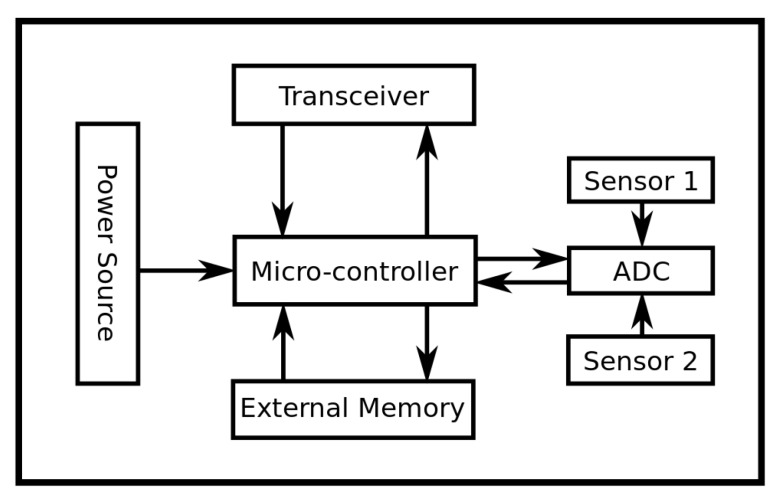
Wireless Sensor Node Schematic.

To verify the comprehensive effectiveness of density control learning method of the wireless sensor mobile node under an IoT environment, an experiment is required as given in [18,19,20,21,22]. In this experiment, the experimental analysis was carried out in Matlab environment. The experimental environment included an IS-3210M CPU, with RAM of 16 GB and Matlab R2010a software. The sensor network nodes with the number of coils of 50 are placed in the square detection area with 100 m edge length, and the radius of the sensor network nodes is set to be 10 m, and the detection area is divided. The transmission broadband between nodes is set to 2 Mbps. in the divided area, 200 nodes are randomly deployed. In this experiment, two kinds of sensor nodes based on CC2430 (represented by A and B) are used to form the sensor network. The monitoring module is responsible for collecting the data. The sensor node is shown in Figure 1.

Our proposed method was compared with the traditional methods given in [5,6,7,8]. In detail, which included an optimized coverage control method for joint sensing wireless sensor [5], a method for grouping control of WSN monitoring nodes based on evolutionary game theory [6], a control method of multi-source fault-tolerant topology node method under wireless sensor actuator network [7] and a control method of three-dimensional WSN topology based on node degree estimation [8]. Experimental results were as follows.

**Figure 2:**
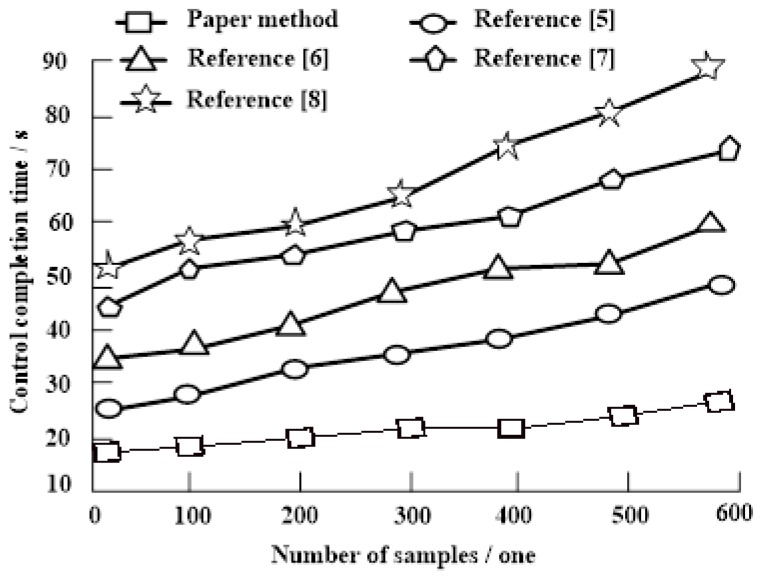
Comparison of node density control completion time.

The comparative experiment of the control completion time(s) was compared based on our proposed method and References [5,6,7,8]. Experimental results are shown in Figure 2.

Figure 2 show that when the number of samples increased, the corresponding network node density control completion time of the five methods increased. When the number of samples increased from 0 to 600, our proposed node density control completion time fluctuated within the range of 17 s~30 s and the fluctuation range was relatively stable. The optimal coverage control method for joint-sensing wireless sensors fluctuated within the range of 22 s~50 s and the fluctuation range showed a slight upward trend. Based on evolutionary game theory, the node control method of WSN monitoring node was controlled to fluctuate within the range of 32 s~58 s and the fluctuation range was increasing. However, the sample number was between 400 and 500 and then continued to rise. A multi-source fault-tolerant topology node control method in a wireless sensor actuator network had a node density control completion time ranging from about 45 s~72 s, and the fluctuation range was increasing. The 3D WSN topology control method based on node degree estimation had a node density control completion time fluctuating within the range of 52 s~90 s. The fluctuation range increased and fluctuated. Experimental results showed that our proposed method had the shortest completion time of node density control, which was better than the other four methods and has higher control efficiency.

The power consumption (Joules) comparison experiment is performed in the method proposed by our proposed method and methods in References [5,6,7,8]. Experimental results are shown in Table 1. In Table 1, Method 1 represented the proposed method; Method 2 represented an optimized coverage control method for a joint-aware wireless sensor; Method 3 represented a clustering control method for WSN monitoring nodes based on evolutionary game theory; Method 4 represented a multi-source fault-tolerant topology node control method in a wireless sensor actuator network; Method 5 represented a three-dimensional WSN topology control method based on node degree estimation.

Table 1 showed that when the number of samples increased, the corresponding network node control power consumption of the five methods increased. Among them, the overall power consumption of this method is less, the maximum power consumption is not more than 25 Joules, and the energy consumption of other methods is up to 70–86 Joules. In addition, the power consumption fluctuation of this method is small. When the number of samples increased from 100 to 400, the control power of the proposed method increased by 5 Joules, and the power of control of an integrated sensing control method for joint sensing wireless sensors increased by 7 Joules. The control power of the group control method increased by 14 Joules based on evolutionary game theory. The control power of the multi-source fault-tolerant topology node control method in the wireless sensor actuator network increased by 13 Joules, and the control power of the 3D WSN topology control method based on the node degree estimation increased by 23 Joules. It can be seen that the power consumption of the proposed method is the smallest and the stability of the control process is good. This paper analyzes the probability of signal interconnection between mobile nodes in the process of analyzing and determining the minimum node density. Therefore, the accuracy of signal interconnection probability calculation between mobile nodes has an important influence on the accuracy of the research results in this paper. The formula for calculating the accuracy of signal interconnection probability between mobile nodes is as follows:

(17) $$G=\frac{C}{J}\times 100\%$$

In Equation (6), *C* represents the node interconnection probability calculated by the method herein, and *J* represents the actual probability of node interconnection. To further verify the effectiveness of this method, the accuracy of signal interconnection probability calculation between mobile nodes is analyzed and compared with References [5,6,7,8] method, as shown in Table 2.

It can be seen from Table 2 that when using this method to calculate the probability of signal interconnection between mobile nodes, the accuracy of the calculation results is about 90%, and the accuracy of Method 2 is not more than 76.3%. The accuracy of Method 3 is relatively high, but it is still lower than that of this method. The accuracy of Method 4 is lower than that of Method 3 and not more than 80.2%. It can be seen that the accuracy of the probability of signal interconnection between mobile nodes is high, which shows that the accuracy of node density control in this method is high. The main reason is that in the process of calculation, any pair of removable nodes are calculated, which has a certain comprehensiveness and improves the accuracy of the results.

## 7. Conclusion

To solve general problems in the current wireless sensor mobile node density control method, such as long control completion time and large power consumption, a density control learning method for wireless sensor mobile nodes in an IoT environment [23,24,25] was proposed. The principle of this method was as follows. Firstly, the relevant hardware IP core in the FPGA development board was configured and the Petalinux system was implemented into it. At the same time, the complex and variable field data processing and control requirements were satisfied by the CC2430 core wireless transceiver module design solution and by using the serial port connection. Then, the advantages of both were combined and the advantages of the WSN were used. Finally, the minimum node density at the time of network connectivity was approximated by exploiting the early and late probability of signal transition between any pair of nodes and by using a normal shadowed regression model and a one-step transition probability matrix. Experimental results showed that our proposed method had a shorter completion time and lower power consumption in comparison to current methods. Lastly, we showed that the node density control accuracy of this method is high, about 90%, which is much higher than that of other methods, which further verifies the superior performance of this method.

## Figures and Tables

**Table 1:** Comparison of different methods of network node control power consumption.

Number of Samples/One	Power Consumption (J)				
	Method 1	Method 2	Method 3	Method 4	Method 5
100	20	63	61	65	63
200	21	65	62	71	69
300	23	69	69	75	72
400	25	70	75	78	86

**Table 2:** Accuracy of signal interconnection probability between mobile nodes based on different methods.

Number of	Calculation Accuracy of Signal Interconnection Probability between Mobile Nodes (%)				
Samples/One	Method 1	Method 2	Method 3	Method 4	Method 5
100	89.8	76.3	80.2	63.5	72.5
200	90.2	74.2	79.5	65.4	74.5
300	91.2	69.3	81.2	69.2	76.1
400	89.3	71.2	83.5	70.2	80.2
